# Putative link between Polo-like kinases (PLKs) and Toll-like receptor (TLR) signaling in transformed and primary human immune cells

**DOI:** 10.1038/s41598-019-49017-z

**Published:** 2019-09-11

**Authors:** Souhayla El Maadidi, Alexander N. R. Weber, Precious Motshwene, Jan Moritz Schüssler, Daniel Backes, Sabine Dickhöfer, Hui Wang, Xiao Liu, Magno Delmiro Garcia, Christoph Taumer, Boumediene Soufi, Olaf-Oliver Wolz, Sascha N. Klimosch, Mirita Franz-Wachtel, Boris Macek, Nicholas J. Gay

**Affiliations:** 1 Interfaculty Institute for Cell Biology, Department of Immunology, Auf der Morgenstelle 15, 72076 Tübingen, Germany; 2 Department of Biochemistry, Cambridge University, 80 Tennis Court Road, Cambridge, CB2 2GA UK; 3 Proteome Center Tübingen, University of Tübingen, Auf der Morgenstelle 15, 72076 Tübingen, Germany; 4 Present Address: University of Pretoria, Agricultural Sciences Building, University & Lynwood rds, Hatfield, Pretoria, 0083 South Africa; 5 Present Address: Department of Epidemiology and Biostatistics, School of Public Health, Nanjing Medical University, No. 818, Tianyuan East Rd, Jiangning District, 211166 Nanjing, China; 6 Present Address: HOT Screen GmbH, Aspenhaustr. 25, 72770 Reutlingen, Germany

**Keywords:** Toll-like receptors, Signal transduction, Chemotherapy

## Abstract

Toll-like receptors (TLRs) are important sentinels of bacterial and viral infection and thus fulfil a critical sensory role in innate immunity. Polo-like kinases (PLKs), a five membered family of Ser/Thr protein kinases, have long been studied for their role in mitosis and thus represent attractive therapeutic targets in cancer therapy. Recently, PLKs were implicated in TLR signaling in mice but the role of PLKs in TLR signaling in untransformed primary immune cells has not been addressed, even though PLK inhibitors are in clinical trials. We here identified several phospho-serine and phospho-threonine residues in the known TLR pathway kinases, Interleukin-1 receptor-associated kinase (IRAK) 2 and IRAK4. These sites lie in canonical polo-box motifs (PBM), sequence motifs known to direct recruitment of PLKs to client proteins. Interestingly, PLK1 was phosphorylated and *PLK* 2 and 3 mRNA induced upon TLR stimulation in primary immune cells, respectively. In whole blood, PLK inhibition disparately affected TLR mediated cytokine responses in a donor- and inhibitor-dependent fashion. Collectively, PLKs may thus potentially interface with TLR signaling in humans. We propose that temporary PLK inhibitor-mediated blockade of TLR-signaling in certain patients receiving such inhibitors during cancer treatment may cause adverse effects such as an increased risk of infections due to a then compromised ability of the TLR recognition system to sense and initiate cytokine responses to invading microbes.

## Introduction

Toll-like receptors (TLRs) are important pattern recognition receptors (PRR) of the innate immune system. In this capacity, TLRs recognize different microbe-associated molecular patterns (MAMPs) but also sense endogenous danger-associated molecular patterns (DAMPs)^1^. Upon activation, TLR signals are integrated and diversified intracellularly by adaptor molecules, one of which is MyD88^1^. Apart from NF-κB, MyD88-dependent signaling is able to activate MAP kinases, interferon regulatory factors (IRFs)^1,2^, and the PI(3) kinase pathway^3^. Through gene transcriptional programs regulated by these pathways important innate immune functions, such as cytokine response or costimulatory molecule expression, are shaped. In humans and mice, four Interleukin-1 receptor (IL-1R)-associated kinase (IRAK) family members, IRAKs 1–4, with non-redundant functions, expression patterns and effector preferences (e.g. early vs late cytokine regulation) are involved in MyD88-dependent downstream signaling^2,4^. IRAKs feature an N-terminal death domain (DD) engaging upstream MyD88 within the so-called Myddosome, an oligomeric post-receptor complex hierarchically assembled via MyD88, IRAK4 and IRAK2 DD protein-protein interactions^5^. Work by us and others showed that naturally occurring loss-of-function mutations in MyD88 prevent Myddosome formation^6^ and suggest that the Myddosome is a critical post-receptor complex in innate immunity^7^. The IRAK kinase domain (KD) activity is switched on first in IRAK4 then IRAK1 or 2 upon Myddosome formation and initiates the subsequent steps of downstream signaling such as the ubiquitination of TNF receptor-associated factor (TRAF) 6. TRAF6 ubiquitination is sensed by the ubiquitin binding regulatory TAB2/TAB3 subunits of the TAK1 complex which subsequently initiates MAPK and IKKα/β activation^8^. One hallmark of the multi-faceted gene transcription programs regulated by the transcription factors activated by MAPK and IKK, namely AP-1 and NF-κB, is the production of pro-inflammatory cytokines. In some cell types, predominantly plasmacytoid dendritic cells (pDC), MyD88-IRAK-dependent signaling activates IFN regulatory factors (IRF) which are important for type I IFN induction. Thus through triggering expression of pro-inflammatory cytokines such as IFNs, TLRs shape courses of infections^1^. Recent data also point to an involvement of MyD88-dependent signaling in the context of non-Hodgkin lymphomas, where *MYD88* gain-of-function mutations promote tumor cell growth^9^. Thus it is of interest to characterize modulators of these signaling events for therapeutic intervention.

Recently an intriguing link between TLR signaling and Polo-like kinases (PLKs) was reported^10^. The human PLK family includes five members, PLK1-5. Especially PLK1 has been extensively studied in the context of cell cycle regulation. In cell cycle regulation, PLK1 cooperates with cyclin-1 dependent kinase 1, cyclin B1 and Aurora kinase and is involved in centrosome maturation, G2/M transition, kinetochore function, mitotic exit and cytokinesis. The roles of other PLKs are more enigmatic but implication as tumor suppressors by interaction with the p53 signaling network have been proposed. PLKs canonically consist of an N-terminal KD and a C-terminal so-called Polo-box domain (PBD)^11^. It is believed that the PBD directs PLKs to substrate molecules by binding to conserved so-called Polo box motif (PBM), typically comprised of Ser-pThr-Pro or Ser-pSer-Pro (SpSP/SpTP)^12^. Binding of the PBD to PBM sequences is then thought to relieve PLK KD inhibition and activate PLK kinase activity. In the context of cell division, many PBM-containing PLK binding partners have been described and proteomics screens identified >600 proteins that interact with PLK1. In the context of cell division, PBM motifs are “generated” through phosphorylation of SSP and STP sequences by cyclin-dependent kinases (CDKs)^13^. Their essential requirement in cell division has given rise to the notion that PLKs constitute attractive targets for cancer therapy (see ref.^14^ and www.clinicaltrial.gov). Inhibition of PLKs resulted in an abortive cell cycle and drive cancer cells into apoptosis^14^. Inhibitors such as BI2536, BI6727 (Volasertib) and GSK461364 have been reported to possess nanomolar IC50 values and target PLK1 KD activity^14^. Fewer approaches have targeted the PBD of PLK1, for example poloxin^14^. The specificity of PBD-targeting inhibitors for PLK1 varies but most inhibitors also affect other PLKs, so that the efficacy of PLK1 inhibitors could at least partially result from additional effects on other PLKs, e.g. PLKs 2 to 4 for which off-target effects of PLK1 inhibitors were reported^14^. Several published academia- and industry-driven phase I and II clinical studies using the abovementioned compounds so far show good tolerability, limited side-effects and promising results in terms of efficacy in non-small cell lung cancer and non-Hodgkin lymphoma^15–17^. Thus PLK inhibitors may be licensed for use in humans at some stage in the near future.

Unfortunately, possibly due to the fact that homozygous PLK1 mice are not viable, relatively little is known about the role of PLK1 in processes other than cell cycle regulation. The same applies to other PLKs. However, a recent report by Chevrier *et al*. identified PLK2 and 4 in an siRNA screen to be involved in TLR signaling in murine cells and PLK1 inhibitors recapitulated the effects observed by PLK2 and 4 knock-down^10^. Specifically, BI2536 blocked transcription of antiviral genes such as *Ifnb1, Cxcl10* and *Cxcl1*. Unfortunately, the situation in human primary immune cells was not addressed. Other groups reported that PLK1 interacted with NEMO (also known as IKKγ) via the scaffold protein TANK (also known as TRAF2) affecting NF-κB signaling in HEK293 and K562 cell lines^18^. Additionally, TANK and IKKβ were found to be phosphorylated by PLK1 in proteomics approaches analyzing samples from Hela or COS7 and HEK293 cell lines, respectively^19,20^. It is unknown which upstream signal(s) prompt(s) the recruitment and/or kinase activity of PLK1 in this context and by which mechanism. Prolyl isomerase (PIN) 1 is another substrate and interactor of PLK1 in Hela cells^19^. PIN1 also interacts with murine IRAK1, and this interaction was dependent on phosphorylation sites in the IRAK1 linker region (S131, S144, S173)^21^. Thus PIN1 may act as a bridging molecule between IRAKs and PLKs. On the other hand, PLKs and IRAKs may counter-influence each other through direct interactions although this has not been proven. Collectively, there is some reported evidence for a role of PLKs in several innate immune signaling pathways. However, all of the above reported data in human cells were exclusively generated using immortalized cell lines and thus may potentially be confounded by continuous PLK activation during cell division. Studies using primary cells have so far only been conducted in murine cells^10^. Therefore it is unclear if PLKs do in fact play a role in human primary immune cells.

After identifying PBMs in members of the IRAK family, we sought to gather evidence for a link between PLKs andTLR mediated responses. Here we report the presence of Polo-box-motifs (PBMs) in human IRAK2 and IRAK4 which would enable a mechanistic link between TLR-MyD88-IRAK signaling and PLKs. PLK1 was phosphorylated upon TLR stimulation and *PLK2* and *3* gene transcription was found to be upregulated in primary human cells. Additionally, we report that PLK inhibitors may interfere with TLR-mediated cytokine production in whole blood in a donor-dependent way.

## Results

### Human IRAK2 and IRAK4 harbor Polo-box motifs

**Figure 1:**
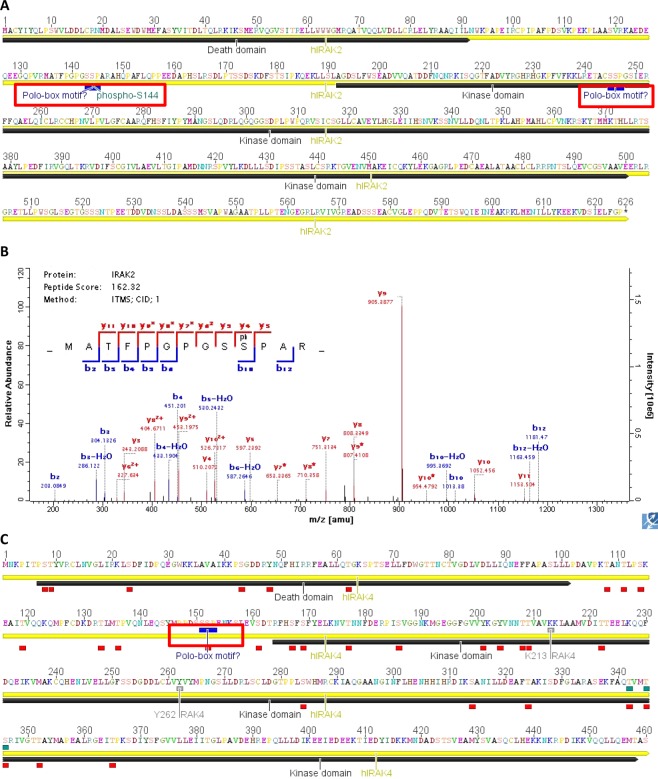
IRAK2 and 4 contain novel phospho-sites that map to Polo-box motifs. (**A**) Overview of human IRAK2 primary sequence (yellow) with DD and KD indicated in dark grey. Putative PBM are in blue with red boxes, and the newly identified phospho-S144 highlighted. (**B**) MS-MS fragment spectrum for the IRAK2 135–147 tryptic fragment containing the S144 phosphorylation (red). (**C**) as in A but shown for human IRAK4. Already published sites are shown in blue, sites newly identified in this study in red. S152 is highlighted.

In a search for regulatory events in TLR pathways, we investigated phosphorylation sites in the human IRAKs and identified phospho- serine 144 (S144) by mass spectrometry of recombinant IRAK2 protein purified from mammalian cells (HEK293T) (Fig. 1A,B). S144 maps to the linker region between the IRAK2 DD and KD (red box in Fig. 1A). The sequence of this linker region is not related to linker regions of the other three IRAK orthologues. Furthermore S144 of IRAK2 is only present in homologues from humans and primates but not in mice. We could also identify several probable auto-phosphorylation sites in IRAK4 after it was purified from *E. coli*, using a buffer composition and ATP concentration that are conducive to the recombinant protein developing kinase activity. This IRAK4 protein was subjected to tryptic digest and analyzed by mass spectrometry(MS). More than 20 new phospho-sites were identified (Fig. 1C) and two of these (S8, T62) when mutated affected the interaction of IRAK4 with MyD88 as shown elsewhere^22^. The newly identified phospho-sites included S152 (red box in Fig. 1C). When inspecting these sites more closely, we noted that pS144 in IRAK2 and pS152 in IRAK4 are part of hitherto unrecognized PBMs, both SpSP (red boxes in Fig. 1A,C). Interestingly, both phospho-sites map to the linker regions connecting DD and KD the structural conformation of which regulates activity in murine Irak1^21^. Primary sequence analysis identified several additional putative PBMs in human IRAKs: in IRAK1 residues 399–401 (S400 is also phosphorylated, not shown); and in the IRAK2 KD at position 275–277 (phosphorylation status of S276 unknown to date, Fig. 1C). To gain a first insight into whether these sites are phosphorylated and possibly regulated in immune cells, we stimulated THP-1 macrophage-like cells with the TLR2 ligand, Pam_2_CSK4, a synthetic deacetylated lipopeptide (here referred to as Pam2), for 30 min and conducted a preliminary phospho-proteomics experiment, analyzing changes in phospho-peptides concerning IRAK1, 2 and 4. Pam2 was chosen as it had previously been shown to affected the phospho-status of IRAK1 S402^23^. As shown in Table 1, IRAK1 phospho-peptides encompassing S399, S401 and S402 (and thus the PBM) were identified, in agreement with independent crystallographic and proteomics evidence^24,25^. In stimulated THP-1 cells the sites changed their abundance between 1,8 to >10-fold following Pam2 stimulation compared to unstimulated cells. For IRAK2, a peptide encompassing S143 and S144 was detected. Even though S143 was downregulated approx. 5-fold upon stimulation, S144 was unaltered, indicating it might be a constitutive, not ligand-dependent PBM. IRAK4 S186, T208 and T345 were also confirmed as phospho-residues but not the putative PBM residue S152. The latter was, however, reported in a comprehensive database of phospho-sites, phospho-site.org, in 5 studies that included human primary cellular material^25–29^. Phospho-site.org generally confirmed all PBM sites described here for IRAKs, except S275 in IRAK2 and S186 in IRAK4, see Table 1. The data from THP-1 cells and comprehensive independent evidence from other studies^23–38^ thus suggest that some, if not all, phospho-sites detected here on overexpressed IRAKs in PBMs are potentially physiologically relevant. Since PBMs had not been noted in IRAKs before and the role of Polo kinases in TLR signaling in human primary cells has not been addressed, we took these findings as a starting point for experimentally exploring a possible connection between PLKs and TLR signaling.

**Table 1:** IRAK1, 2 and 4-related phospho-peptides.

Gene names	Protein Uniprot ID	Position in peptide sequence	Localization probability	PEP	Number Phospho-STY sites	Amino acid	Sequence and Phospho (STY) Probabilities	Position in peptide	Charge	Mass error [ppm]	Ratio treatment/untreated	Listed in phospho-site.org?
*IRAK1*	D3YTB5-1	399	0,82	1.92E-13	1	S	FAGSS(0.004)PS(0.816)QS(0.144)S(0.035)MVAR	7	2	*−16,24*	*1,86*	*YES (S373)* ^24^
*IRAK1*	D3YTB5-1	401	0,90	3.24E-40	1	S	FAGSSPS(0.002)QS(0.902)S(0.095)MVAR	9	2	*−17,26*	*13,30*	*YES (S375)* ^24, 25^
*IRAK1*	D3YTB5-1	402	0,88	2.56E-62	1	S	FAGSSPS(0.002)QS(0.114)S(0.883)MVAR	10	2	−0,41	11,71	*YES (S376)* ^23– 25^
*IRAK1*	D3YTB5-1	413	0,73	0,000344	2	T	TQTVRGT(0.726)LAY(0.637)LPEEY(0.637)IK	7	2	-0,63	0,48	*YES (T387)* ^23, 24, 37, 38^
*IRAK2*	O43187	143	0,70	2.15E-14	1	S	MATFPGPGS(0.696)S(0.304)PAR	9	2	0,11	0,24	YES (S143)^31^
*IRAK2*	O43187	144	0,50	0,00182		S	MATFPGPGS(0.5)S(0.5)PAR	10	2	*33,36*	*n/a*	YES (S144)^25,30^
*IRAK4*	Q9NWZ3-1	152	n/a	n/a	n/a	n/a	n/a	n/a	n/a	n/a	n/a	*YES* ^25– 29^
*IRAK4*	Q9NWZ3-1	186	1,00	1.53E-10	1	S	NVTNNFDERPIS(1)VGGNK	12	3	*12,67*	*20,97*	*NO*
*IRAK4*	Q9NWZ3-1	208	0,94	4.64E-06	1	T	GYVNNT(0.937)T(0.063)VAVK	6	2	*27,17*	*n/a*	*YES (T208)* ^29^
*IRAK4*	Q9NWZ3-1	345	0,89	1.81E-13	1	T	FAQT(0.021)VMT(0.887)S(0.092)R	7	2	-0,71	0,95	YES (T345)^29,30,32–36^

THP-1 macrophages previously labeled with stable isotopes were treated with Pam2 (5 μM) for 30 min, lysates prepared and the containing phospho-peptides analyzed by Mass spectrometry (see Methods). A single discovery/screening experiment was done. When sites were also listed on https://www.phosphosite.org this was also indicated below with residue numbering given in brackets.

### PLKs are transcribed upon TLR stimulation

**Figure 2:**
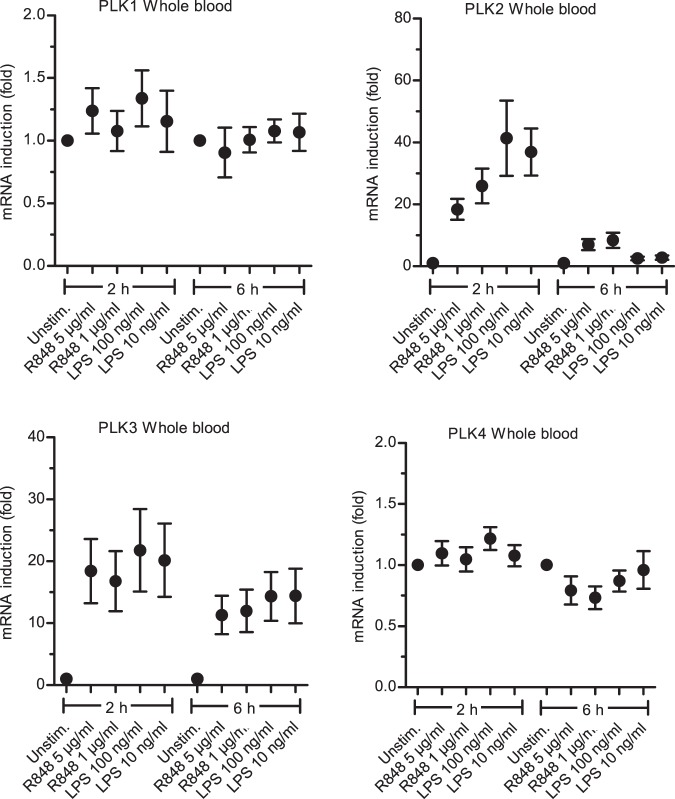
TLR stimulation induces *PLK2* and *3* mRNA expression. Whole blood was treated with R848 or LPS at the indicated concentrations and for 2 or 6 h. Subsequently, total RNA was isolated, genomic DNA digested, reverse-transcribed and used in quantitative real-time PCR for the indicated PLKs relative to the TBP housekeeping gene. Four donors were assayed in triplicates. Biological means +/− SEM are shown.

Since many TLR pathway signal transducers such as MyD88 are themselves regulated by TLR signaling, we tested whether PLKs were also regulated at the mRNA level in whole blood. Figure 2 shows that treatment of freshly drawn human whole blood with the TLR7/8 agonist R848 or the TLR4 agonist LPS induced cellular *PLK2* and *PLK*3 mRNA levels by approximately 20-fold or 10-fold within 2 h, respectively. For *PLK3*, mRNA induction remained high up to 6 h whereas *PLK2* induction decreased by 6 h. *PLK1* and *PLK4* were not induced by TLR stimulation. Likewise the TLR3 ligand poly I:C and IL-1 did not induce PLK transcription. This suggests that PLKs may be part of a TLR-elicited feed-forward loop relevant for effector outcomes or to anticipate cellular proliferation.

### PLK1 is phosphorylated upon TLR stimulation

**Figure 3:**
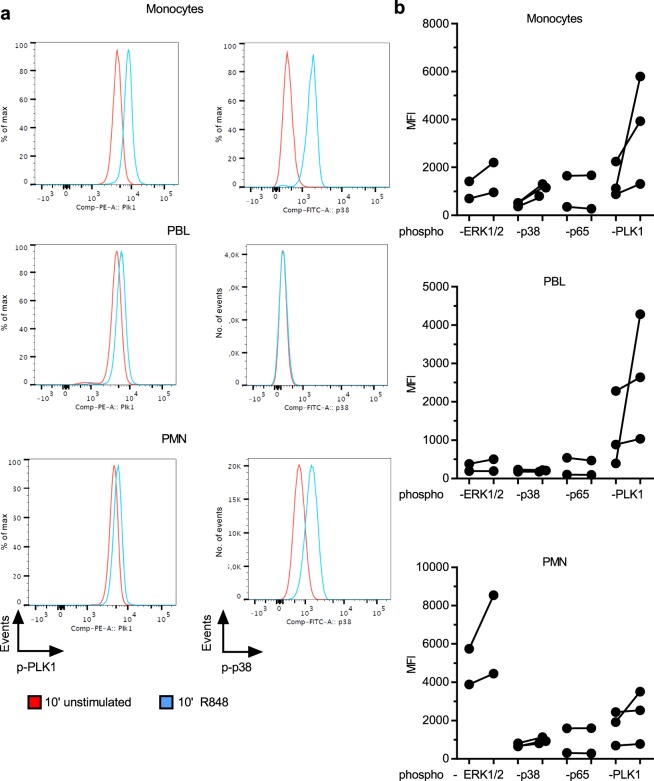
PLK1 is phosphorylated in primary immune cells upon TLR stimulation. Freshly drawn whole blood was treated with R848 or left untreated for 10 min before erythrocytes were lysed, cells fixed, permeabilized and stained using anti-phospho-PLK1, -p38, -ERK1/2 or –p65. Gates were set to distinguish monocytes, peripheral blood lymphocytes (PBL) and PMN as shown in Fig. S1. Event # are given as indicated. In (**a**) one representative donor is shown, in (**b**) data from 2–3 donors are summarized. Each dot represents one donor. Differences were not statistically significant by one-way ANOVA with Sidak correction for multiple testing due to donor-to-donor variations and the low number of donors.

We next sought to determine whether PLKs are directly activated in response to TLR signaling as well as being transcriptionally upregulated. During mitosis, phosphorylation of PLKs is an indicator of activation^39^. Since there are currently no phospho-specific antibodies for the highly regulated PLKs 2 or 3, we focused on the modification of PLK1 as a representative of the PLK family that has been widely characterized and for which phospho-specific antibodies are available. Whole blood was thus analyzed by anti-phospho-Thr210 flow cytometry^40^ after 10 min of R848 stimulation. Figure 3 shows that PLK1 was phosphorylated in gated monocytes, lymphocytes (PBL) and neutrophils (PMN), with monocytes showing the greatest differences in MFI for all donors (n = 3, Fig. 3b). Although further mechanistic details remain to be elucidated, these data suggest that at least PLK1 is activated by TLR triggering and may thus contribute to TLR signal transduction by kinase activity or scaffolding function.

### Inhibition of PLK in human primary immune cells disparately affects cytokine induction

**Figure 4:**
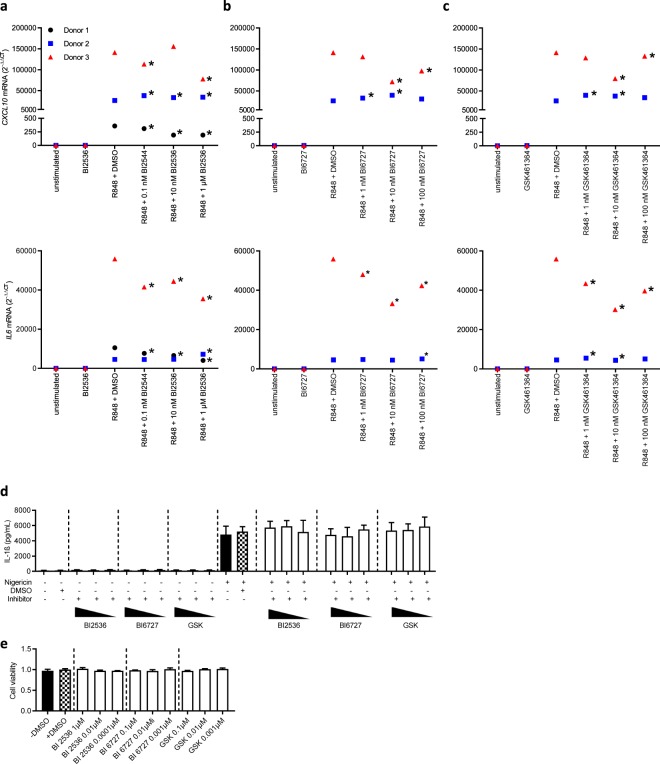
PLK inhibitors BI2536 and BI6272 donor-independently modulate TLR- but not NLRP3 inflammasome-dependent cytokine responses. Freshly drawn whole blood was treated with 5 µg/ml R848 in the presence or absence of the indicated concentrations of BI2536 (**a**), BI6272 (**b**) or GSK461364 (**c**), respectively, for 3 h or left unstimulated. Subsequently, total RNA was isolated, genomic DNA digested, reverse-transcribed and used in quantitative real-time PCR for *IL6* and *CXCL10* mRNA relative to *TBP*. Each sample was measured in triplicates and individual symbols represent the mean for each donor coded by symbol type and color. The effects of inhibition were tested with reference to R848 + DMSO control using an unpaired two-tailed t-test. Technical triplicates that were statistically significantly (p < 0.05) higher or lower than the donor’s R848 + DMSO are marked by *. A combined statistical analysis did not yield significant differences due to high inter-individual variation. (**d**) ELISA analysis of IL-1ß in supernatants from PMA-differentiated THP-1 cells treated for 1 h with the indicated PLK1 inhibitors and then stimulated with the NLRP3 agonist, nigericin. (**e**) Cell viability using CCK8 reagent normalized to DMSO control. In D and E data are representative of two independent experiments.

The data so far suggested that PLKs are regulated duringTLR signaling. If indeed this was the case, PLK inhibition may affect TLR mediated cytokine transcription. Given the reported observation for a role of PLK in murine innate immune responses, we sought to test if pharmacological inhibition of PLK kinase activity using BI2536, BI6727 or GSK461364^41^ affected the induction of cytokines in response to R848. As shown in Fig. 4, stimulation of whole blood from healthy donors with R848 resulted in robust induction of *IL6* and *CXCL10* mRNA, albeit with high inter-individual variation. Although the effects were moderate and differed between donors, the application of BI2536 led to a dose-dependent reduction in both *CXCL10* and *IL6* mRNA levels in 2/3 of the donors, in some instances at an inhibitor concentration as low as 1 nM (Fig. 4a). BI6272 (Fig. 4b) and GSK461364 (Fig. 4c) were only analyzed in two donors, donor 2 and 3. Here donor 3 showed an inhibition of cytokine induction for both PLK inhibitors. Donor 2 remained refractory to all inhibitor treatments. Of note, PLK1 inhibitors did not affect another important innate immune signaling machinery, the NLRP3 inflammasome^42^: this was evident when THP-1 cells were treated with PMA and Nigericin, which triggers IL-1β release in these cells^43^. Conversely to a Bruton’s Tyrosine Kinase (BTK) inhibitor^44^, PLK inhibitors BI2536, BI6272 and GSK461364 did not block IL-1β release (Fig. 4d) which is regulated transcriptionally by PMA or TLR ligand, and maturation via the NLRP3 inflammasome^45^. Cellular toxicity upon PLK1 inhibition was not observed (Fig. 4e). Although further donors will therefore need to be assessed, our results thus suggest a putative contribution of PLKs to TLR-dependent cytokine responses in certain donors.

## Discussion

Over the last decades there has been much interest in exploring PLKs as targets in cancer therapy^14^. The data currently available suggests PLK inhibitors are efficacious in patients. This has been mainly attributed to the effects PLKs have on tumor cells themselves, i.e. the blocking of cell division. On the other hand, the effect of PLK inhibition on non-transformed cells has so far received little attention. Chevrier *et al*. recently proposed a role for murine PLKs in antiviral TLR signaling, posing the question whether PLK inhibition in humans might compromise innate immune defenses. If so, this should best be anticipated during phase III trials or later clinical use of PLK inhibitors. However, this previous study only focused on murine primary cells and cell lines and all other data in the human system have only been gleaned from transformed cells in which PLKs are likely to be regulated differently. Our observations in human primary whole blood, a highly physiological system for assessing immune activity, suggest that PLK inhibitors may interfere with TLR-mediated cytokine production in certain subjects. Specifically and in keeping with the data by Chevrier *et al*., *CXCL10* expression was reduced by PLK inhibition in response to TLR agonists^10^. Generally, a sufficient cytokine response is required for efficient immune cell recruitment to sites of infection and the mounting of an adaptive immune response. Thus, PLK inhibition in patients may entail immunosuppression which is a phenomenon observed for many chemotherapeutic drugs, e.g. inhibitors of BTK, a signaling molecule acting downstream of B cell receptor and TLR signaling pathways^46,47^ which we recently found to be a direct regulator of the NLRP3 inflammasome^44^. Our data suggest that PLK inhibitors may compromise innate immunity, potentially via a direct effect on TLR signal transduction. Mechanistically, the suppression of PLK kinase activity may have a negative impact on phosphorylation events that promote signaling. At this point we can only speculate whether this will directly affect IRAKs or other pathway members and whether this occurs by PLKs being associated with IRAKs either constitutively or only upon TLR signaling. Apart from a role as kinases, it is conceivable that PLK operate as scaffolds that reinforce interactions in post-receptor signaling. The effect of different TLR agonists on PLK1 phosphorylation, the inhibition conferred by PLK blockers, and the occurrence of PBMs in several IRAKs kinases would support the notion that PLKs connect and potentially operate on components of the Myddosome, where TLR pathways converge^6^. IRAKs and PLKs both were shown to separately interact with the prolyl-isomerase PIN1^21,48^, which is also a PLK substrate. A direct interaction or substrate-kinase relationship between endogenously expressed IRAKs and PLKs has, however, yet to be demonstrated are significant limitations of this study. Since co-overexpression and co-immunoprecipitation in transformed cells, e.g. in HEK293T cells may be confounded by overexpression artefacts, absence of endogenous signaling events or repetitive cell cycling, we attempted to co-immuno-precipitate PLKs and IRAKs from primary monocytes. Unfortunately, this failed in our hands due to the unavailability of specific PLK antibodies to endogenous PLKs Thus further mechanistic work thus remains clearly warranted in future, e.g. studying the effect of the newly identified PBM phospho-sites in IRAK2 and IRAK4 on PLK interactions and TLR post-receptor signaling: Discovering both the kinase(s) phosphorylating the PBMs in IRAKs, as well as substrated phosphorylated by PLKs after recruitment via these phosphorylated PBMs would be interesting next steps. That IRAK phosphorylation may critically regulate TLR post-receptor signaling at the level of the Myddosome complex^6,49^ was recently shown by our groups for serine 8 in IRAK4^22^. In depth studies of the novel IRAK phospho- and PBM sites identified here may thus provide further insights into how TLR signaling is regulated and how PLK inhibitors may interfere with the TLR arm of innate immunity. Elucidating the latter appears mandatory to anticipate the potential impact on innate immunity in patients undergoing PLK inhibitor treatments. Although the number of donors analyzed for the inhibitor study was very limited and needs to be expanded (ideally to healthy donors and cancer patients) before drawing more generally applicable conclusions, our results suggest that suppression of TLR signaling could be an unwanted side-effect for the use of PLK inhibitors in humans, they at the same time open up the possibility that PLK inhibitors may offer therapeutic opportunities under disease conditions where excessive TLR-driven cytokine production is pathophysiological, for example in rheumatoid arthritis, psoriasis or lung inflammation Further investigations in experimental *in vivo* models or monitoring the effects on TLR signaling in clinical studies are clearly warranted.

## Methods

### Reagents and cells

Reagents were from Sigma unless otherwise stated. The following TLR ligands were used: Pam_2_CSK_4_ (Axxora or Invivogen), poly(I:C) (Sigma), R848 (Invivogen), LPS (Invivogen). Cellular viability was determined using the CCK8 reagent (Dojindo) according to the manufacturer’s instructions.

### IRAK2-HA expression and purification

Human IRAK2 with StrepHA tag^50,51^ was expressed from HEK 293 FlipIn TRex as outlined in supplemental methods. In brief, IRAK2-StrepHA-containing lysates were loaded onto a Strep-tactin Sepharose (IBA) column, washed 3 times and eluted with Buffer E (100 mM Tris, pH 8.0; 150 mM NaCl, 1 mM EDTA, 2.5 mM des-thiobiotin). Elution fractions were checked on silver stained gel for protein yield and purity (verified by SDS-PAGE followed by standard silver staining) and pooled for MS/MS.

### Mass spectrometry analysis of IRAK2

Protein samples were dissolved in denaturation buffer (DB), reduced and alkylated before LysC (Waco) digestion. Phospho-peptides were enriched using TiO_2_ beads, subjected to liquid chromatography on an EasyLC nano-HPLC (Proxeon Biosystems) with a nano-HPLC column with C18 spheres (Dr. Maisch) and MS/MS analysis performed on a LTQ-Orbitrap XL mass spectrometer (Thermo Fisher Scientific). Further details are provided in Supplemental Methods.

### IRAK4 expression and purification

Human IRAK4 was cloned into the pETG30 vector and expressed in BL21 Codon Plus cells as previously described^22,49^. Briefly, IRAK4 was expressed as a GST fusion protein and Prescission protease was used to cleave it from its fusion partner. It was thereafter purified by both ion exchange and size exclusion chromatography.

### Dimethyl-labeling of THP-1 cells and global phosho-proteome mass spectrometry analysis

THP-1 cells (Invivogen) were primed with PMA (300 ng/mL, InvivoGen) overnight and then stimulated with PBS or 5 µM Pam2 for 30 minutes. Dimethyl-labeling of equal amounts of lysate, sample workup, MS acquisition, peak annotation and database searches was done as described in Supplemental Methods. Data were deposited in the PRIDE database (identifier PXD007542).

### NLRP3 inflammasome analysis

THP-1 cells were primed with PMA (100 ng/mL, InvivoGen) overnight, incubated with PLK1 inhibitors BI 2536, BI 6727, GSK461364 (Selleckchem, at 1, 0.01 or 0.001 μM) for 1 hour, and then stimulated with 15 µM nigericin (InvivoGen) for 1 h. IL-1ß levels in supernatants were determined with half-area plates by using ELISA (BioLegend) with quadruplicate points on a standard plate reader. Cell viability was assayed using Cell Counting Kit-8 (CCK-8, Dojindo Laboratories) following the manufacturer’s instructions. The absorbance at 450 nm was measured using a microplate reader.

### Whole blood analysis of cytokine transcription and phospho-flow

Heparinized whole blood was drawn from healthy volunteers at the Department of Immunology, Tübingen upon written informed consent and according to a study protocol approved by the ethics committee of the Medical Faculty of Tübingen University, Germany. Phospho-flow analysis Cytokine transcription in whole blood was done as described before^40,50^ and detailed in Supplemental Methods.

### Real-time PCR

mRNA was isolated (cell lines: RNeasy Mini Kit; whole blood: QIAamp RNA Blood Mini Kit; Qiagen) and transcribed to cDNA (High Capacity RNA-to-cDNA Kit; LifeTechnologies). The expression *IL6* (Hs00985639_m1) and *CXCL10* (Hs01124251_g1) was studied using the indicated TaqMan Gene Expression Assays (LifeTechnologies). Data were normalized to the housekeeping gene TBP (Hs00427621_m1; LifeTechnologies). PLK1-4 primers for SYBR Green qPCR are listed in Supplementary Methods. The samples were analyzed on a real time cycler (Applied Biosystems; 7500 fast).

### Statistical analysis

For functional analyses, data was analyzed using Excel 2010 (Microsoft) and GraphPad Prism 5.0 (GraphPad Software, Inc.). p-values were determined using an unpaired *t* test, as indicated. p-values < 0.05 were generally considered statistically significant and are denoted by * throughout.

## Supplementary information

Supplemental methods, Figure S1 and Table S1
