# Supplementary material for: Putative link between Polo-like kinases (PLKs) and Toll-like receptor (TLR) signaling in transformed and primary human immune cells
Source: Sci Rep. 2019 Sep 11;9:13168. doi: 10.1038/s41598-019-49017-z (PMC6739412; doi:10.1038/s41598-019-49017-z)
Supplement: Supplementary file 1 — Supplemental methods, Figure S1 and Table S1 [file 41598_2019_49017_MOESM1_ESM.docx]

**Supplemental information**

Title

**Putative link between Polo-like kinases (PLKs) and Toll-like receptor (TLR) signaling in transformed and primary human immune cells**

**Authors**

Souhayla El Maadidi^&a^, Alexander N. R. Weber*^&a^, Precious Motshwene^b$^, Jan Moritz Schüssler^a^, Daniel Backes^a^, Sabine Dickhöfer^a^, Hui Wang ^a$^, Xiao Liu^a^, Magno Delmiro Garcia^a^, Christoph Taumer^c^, Boumediene Soufi^c^, Olaf-Oliver Wolz^a^, Sascha N. Klimosch^a$^, Mirita Franz-Wachtel^c^, Boris Macek^c^, Nicholas J. Gay*^b^

**Supplemental methods**

*Reagents and cells*

Reagents were from Sigma unless otherwise stated. The following TLR ligands were used: Pam_2_CSK_4_ (Axxora or Invivogen), poly(I:C) (Sigma), R848 (Invivogen), LPS (Invivogen).

*IRAK2-StrepHA expression and purification*

An expression construct for human IRAK2 with StrepHA tag [^1^](#_ENREF_1)^,^[^2^](#_ENREF_2) was stably integrated into HEK 293 FlipIn TRex grown in Tetracycline-free media. The expression was induced in 20 x 175-cm² cell culturing flasks at 75% confluency by the addition of 1 µg/ml doxycycline for another 24 h. The media was then aspirated, the cells washed with 5 ml pre-warmed PBS, and detached for 20 min at 37 °C using PBS with 0.5 mM EDTA. Cells were washed off from flask bottom with the containing PBS-EDTA and transferred to a 50 ml centrifuge falcon tube (BD). Flasks were rinsed with 5 ml additional PBS-EDTA, which was also transferred to the same falcon tube. Falcon tubes were centrifuged at 1800 xg, 5 min, without brake. The supernatant was discarded, the pellet was washed twice with warm PBS. After aspiration of the wash buffer, the pellet was first dislodged and dissociated and thecell suspension was snap-frozen in liquid nitrogen and stored at - 80 °C until lysis and purification. For lysis, the pellet was thawed on ice and lysed in 2 ml TNN-HS buffer (IBA). The pellet was thoroughly pipetted up and down for lysis. The lysate was incubated for 20 min on ice then vortexed 3 times and centrifuged (20 min, 18.000 rpm, 4 °C, no brake). Supernatant was kept and centrifuged again. An aliquot for SDS-Page was kept and the rest of supernatant was filtered (0.45 µm). An aliquot of filtered lysate was kept. 50 % slurry of Strep-tactin Sepharose (IBA) was added to a 10 ml column forming a bed volume of 6 ml. Column was washed 3 times with 2 bed volumes (BV) of Buffer W (100 mM Tris, pH 8.0; 150 mM NaCl, 1mM EDTA). Beads were resuspended in 2 BV TNN-HS buffer and were transferred to a 50 ml falcon containing the cell lysate. The Falcon tube was rotated for 30 min and then the lysate containing the resin was transferred back into the column. The resin was allowed to settle and the lysate was drained off. The Flow-through was collected. The column was then washed with 5 x 2 BV buffer W (IBA). Subsequently, Buffer E (100 mM Tris, pH 8.0; 150 mM NaCl, 1mM EDTA, 2.5 mM des-thiobiotin) was added for eluting IRAK2-StrepHA, and 500 µl factions were collected in 1.5 ml Eppendorf tubes. These fractions were checked on silver stained gel for protein yield and purity (verified by SDS-PAGE followed by standard silver staining) and pooled for MS/MS.

Mass spectrometry analysis of IRAK2

Protein samples were dissolved in denaturation buffer (DB) at a concentration of ~1-2 µg/µl. Reduction buffer was added to a final concentration of 1 mM dithiothreitol (DTT) and samples were incubated for 1 h at RT. Alkylation buffer was added to a final concentration 5.5 mM iodacetamide (IAA) and incubated at RT in the dark for 1 h. pH was checked to be 8.0 and adjusted if necessary with Trifluoroacetic acid (TFA)*.* 1 µg of lysyl endopeptidase LysC (Waco) was added per 100 µg of protein and incubated for 3 h at RT. Samples were diluted with 4 volumes of distilled water. pH was checked to be 8.0 and adjusted if necessary with TFA. 1 µg Trypsin was added per 100 µg protein and incubated overnight at RT. Reaction was stopped by acidifying samples with TFA to a maximal concentration of 0.1 %.

To the digested samples, acetonitrile was added to a final concentration of 30 % and pH was adjusted to 2-3. 5 mg of TiO_2_ beads were mixed with 50 µl of loading solution (30 mg/ml 2,5 dihydrobenzoic acid (DHB; Fluka), 80 % acetonitrile in water) and incubated for 10 min at RT. TiO_2_ solution was added to sample and incubated for 30 min at RT in an orbital shaker. Sample was then centrifuged at 13,000 rpm for 2 min. Supernatant was removed. Beads were washed in 1 ml Solution I (30 % ACN, 3 % TFA) for 10 minutes on a shaker followed by a centrifugation step and discarding the supernatant (see above). Beads were then washed in Solution II (80 % ACN, 0.1 % TFA) for 10 min on a shaker. Samples were again centrifuged, supernatant was discarded. Beads were resuspended in 50 µl Solution II and transferred to a pipet tip, packed with C8 material. A hole was pierced through the lid of a 2 ml Eppendorf tube, and the tip was pushed through. Eppendorf tube with tip was centrifuged for 3 min at 5000 rpm (Biofuge Pico), flow-through was discarded. Elution was realized by pushing the pipet tip with TiO_2_ beads into a new Eppendorf tube, adding 100 µl 40 % ammonia solution (25 % in water) in CAN (pH 10.5) and centrifuging for 3 min at 5000 rpm. Elution step was repeated twice adding to a final eluate of 300 µl. Eluate was stage tipped and handed over to PCT for MS-Analysis.

For liquid chromatography an EasyLC nano-HPLC (Proxeon Biosystems) with a nano-HPLC column (75 µm inner diameter, length 15 cm), self-packed with 3 µm C18 spheres (Dr. Maisch, Ammerbuch, Germany) was used. Column was loaded at a flow rate of 500 nL/min with peptide mixture suspended in HPLC solvent A (0.5 % acetic acid), without pre-column. The peptide mixture was eluted at a constant flow rate of 200 nL/min with a linear, segmented gradient of 5–33 % HPLC solvent B (80 % acetonitrile in 0.5 % acetic acid).

MS/MS analysis was performed on a LTQ-Orbitrap XL mass spectrometer (Thermo Fisher Scientific). Ionization was realized by an electrospray ion source (ESI) (Proxeon Biosystems). The mass spectrometer operated in positive ion mode with an acquisition time of 150 min per run. The acquisition cycle started with one initial full scan of the injected peptides in the Orbitrap analyzer (MS) followed by the fragmentation by collision induced dissociation (CID) of the 5 most intense multiple charged precursor ions and a rescan (MS/MS) in the linear ion trap analyzer (LTQ). A full scan had a scan range of m/z 300-2000 at a resolution (R) of 60,000. For real-time recalibration of MS spectra, the lock-mass option was applied. Dynamic exclusion of already fragmented ions in the past 90 sec was performed in order to suppress high abundant peptide ions. Multistage activation (MSA) was enabled for phospho-analysis in all MS/MS events with the neutral loss masses of singly (‑97.97 Th), doubly (‑48.99 Th), and triply (‑32.66 Th) charged precursor ions. Optionally, an inclusion list was programmed, preferring specifically defined precursor ions over the most intense ones. Mass spectrometry was conducted by the Proteome Center Tübingen (PCT).

In the first experiment, both TiO_2_-enrichment and an inclusion list were omitted and the overall coverage of IRAK2-StrepHA was: 56.1 %. One phospho-site was discovered but could not be localized. Localization was narrowed to be either on Serine 143 or Serine 144. In a second mass-spectrometry experiment TiO_2_ phospho-peptide enrichment was conducted prior to LC-MS/M and an inclusion list of most likely phosphorylated residues (detected peptides of the first MS-run and predictions based on netPhos scores (> 0.95), conservation of phosphosite in other members of the IRAK family an localization of the possible site at the surface of IRAK2 assessed by PyMol). The theoretical produced peptides by tryptic digest of IRAK2 were calculated with protein prospector, conditions were set to peptides at least charged double positive and the resulting masses had to be in scan range of the mass spectrometer. This resulted in a list of 60 masses, taking the following residues into account: S144, S143, T207, S249, S488, T487. The LC-MS/MS measurement resulted in an overall coverage of 59.04 % (in silico digested peptides in range of the mass spectrometer: 98.56 % = theoretical possible coverage). A phosphosite localizing to position S144 was confirmed with low PEP score and high localization probability. Properties of the identified phosphosite are listed in supplemental information and the fragment spectrum shown in Fig. 1D.

IRAK4 expression and purification

Human IRAK4 was cloned into the pETG30 vector and expressed in BL21 Codon Plus cells as previously described [^3^](#_ENREF_3)^,^[^4^](#_ENREF_4). Briefly, IRAK4 was expressed as a GST fusion protein and Prescission protease was used to cleave it from its fusion partner. It was thereafter purified by both ion exchange and size exclusion chromatography.

Dimethyl-labeling of THP-1 cells and global phosho-proteome mass spectrometry analysis

THP-1 cells (Invivogen) were grown in complete RPMI medium and differentiated with 300 ng/ml PMA for overnight. The next day the medium was exchanged to PMA free complete media and the cells rested for another 48 h before stimulation with PBS or 5 µM Pam2 for 30 minutes. After washing with ice-cold PBS (containing phosphatase and protease inhibitors, Roche), cells were lysed in 800 µl of lysis buffer (6 M guanidinium hydrochloride, 5 mM Tris(2-carboxymethyl)phosphine, 10 mM chloroacetamide, 100 mM Tris-HCl pH 8.5) heated to 99°C for lysis, reduction and alkylation of proteins. Cells were incubated for 10 minutes at 99°C, sonicated for 10 minutes and proteins digested with Trypsin overnight at room temperature. Protein concentration was determined using a Bradford assay. 7.9 mg total lysate were desalted on Sep-Pak C18 cartridges and dimethyl-labeled as published [^5^](#_ENREF_5). Prior mixing of protein samples, label incorporation and proper mixing was checked. The sample was fractionated on an offline Ultimate 3000 liquid chromatography system equipped with xBridge BEH C18 130A, 3.5 µm, 4.6 x 250 mm column operated under high pH using 5 mM NH_4_OH buffers. 60 fractions were collected and each fraction was analyzed by LC-MS/MS for full proteome analysis. The 60 fractions were concatenated into 15 pools and dried by vacuum centrifugation. Peptide pools were reconstituted in 1 ml of 80% ACN, 6% TFA and enriched with TiO_2_ spheres for 30 minutes in a protein to bead ratio of 2:1. Pelleted beads were washed with 100µl of 30% ACN, 1% TFA followed by 80% ACN, 1%TFA. Elution from the beads was performed two times with 100 µl of 5% ammonia hydroxide solution in 60% acetonitrile (pH > 10.5) and once with 10 µl 1% formic acid in 80% ACN. Acetonitrile was eliminated by vacuum centrifugation and phospho-peptides desalted on Stagetips prior LC-MS/MS measurements. LC-MS/MS analyses were performed on an Easy-nLC 1200 equipped with a 20 cm and 75 µm ID PicoTip fused silica emitter packed with ReproSil-Pur C18-AQ 1.9 µm resin (Dr. Maisch Ltd.) coupled to an Q Exactive HF (both Thermo Scientific). Phospho-peptide enriched samples were separated using a 87 minute segmented gradient of 5-50% solvent B (80% ACN in 0.1% formic acid) at a constant flow rate of 200 nl/min while high pH LC fractions were separated by an 35 minute gradient. The Q Exactive HF was operated in positive mode. Full scans were acquired from m/z 300 to 1,650 with a resolution of 60,000. For proteome measurements the 20 most intense ions and for phospho-proteome the seven most intense ions were fragmented by higher energy collisional dissociation (HCD) and MS/MS spectra recorded with a resolution of 30,000 and 60,000, respectively. The MS data were processed using default parameters of the MaxQuant software suite (v1.5.5.1) [^6^](#_ENREF_6). Extracted peak lists were submitted to database search using the Andromeda search engine [^7^](#_ENREF_7) to query a target-decoy database of *H. sapiens* proteome (release 2014_02; 88,692 entries). In database search, full tryptic specificity was required and up to two missed cleavages were allowed. Carbamidomethylation of cysteine was set as fixed modification; protein N-terminal acetylation, oxidation of methionine, and phosphorylation of serine, threonine, and tyrosine were set as variable modifications. Overall light-, medium- and heavy- demethylation labeling on lysine residues and peptide N-termini were defined. “Re-quantify”, calculating ratios for isotope-patterns not assembled into labeling triplets, was enabled. Initial precursor mass tolerance was set to 4.5 ppm at the precursor ion and 20 ppm at the fragment ion level. False discovery rates were set to 1% at peptide, phosphorylation site, and protein group level. The MaxQuant output was analyzed using the Perseus software (1.5.3.2). Pearson correlation coefficient was calculated for replicates. Differentially expressed proteins and phosphorylation events were identified using the two-tailed, signal intensity-weighted “Significance B” test (p ≤ 0.01). Proteome and phospho data were mapped to pathways using the KEGG Pathway Mapper (<http://www.genome.jp/kegg/tool/map_pathway2.html>) and manually annotated regarding the regulation of individual proteins or phospho-sites. The mass spectrometry proteomics data have been deposited to the ProteomeXchange Consortium via the PRIDE partner repository at [www.ebi.ac.uk/pride](http://www.ebi.ac.uk/pride) with the dataset identifier PXD007542.

NLRP3 inflammasome analysis

THP-1 cells were primed with PMA (100 ng/mL, InvivoGen) overnight, overnight, incubated with PLK1 inhibitors BI 2536, BI 6727, GSK461364 (Selleckchem, at 1, 0.01 or 0.001 μM) for 1 hour, and then stimulated with 15 µM nigericin (InvivoGen) for 1 h. IL-1ß levels in supernatants were determined with half-area plates by using ELISA (BioLegend) with quadruplicate points on a standard plate reader. Cell viability was assayed using Cell Counting Kit-8 (CCK-8, Dojindo Laboratories) following the manufacturer's instructions. The absorbance at 450 nm was measured using a microplate reader.

Whole blood analysis of cytokine transcription and phospho-flow

Heparinized whole blood was drawn from healthy volunteers at the Department of Immunology, Tübingen upon written informed consent and according to a study protocol approved by the ethics committee of the Medical Faculty Tübingen. For the analysis of cytokine transcription whole blood was treated as indicated and incubated at 37°C and 5% CO_2_. Cells were harvested for RNA isolation and qPCR analysis 3 h post stimulation with TLR agonists as described in [^8^](#_ENREF_8). Phosflow analysis on stimulated whole blood was carried out as described [^8^](#_ENREF_8). In brief, freshly drawn whole blood was treated as indicated and incubated for ten minutes at 37°C in a water bath. Red blood cells were lysed and leukocytes were fixed in one step (Lyse/Fix Buffer, BD). LIVE⁄DEAD Fixable Aqua was used to stain dead cells (Life Technologies) before the cells were permeabilized with methanol for ten minutes on ice. Fc-receptors were blocked with Flebogamma (50 µg/ml, Grifols Biologicals) and cells were stained with phospho-specific antibodies (ERK1/2 pT202/pY204 #612593; p38 MAPK pT180/pY182 #612594; p65 pS529 #558423, all from BD) or surface marker (CD33 PerCP-Cy5.5 (BD #333146) for 45 minutes at 4°C. Anti-Phospho-PLK1 (Thr210) (clone D5H7) was from CST. A standardized protocol and identical flow cytometer (FacsCanto II, BD) settings were always used for all donors. Further settings on request.

Real-time PCR

mRNA was isolated (cell lines: RNeasy Mini Kit; whole blood: QIAamp RNA Blood Mini Kit; Qiagen) and transcribed to cDNA (High Capacity RNA-to-cDNA Kit; LifeTechnologies). The expression *IL6* (Hs00985639_m1) and *CXCL10* (Hs01124251_g1) was studied using the indicated TaqMan Gene Expression Assays (LifeTechnologies). PLK1-5 mRNA was measured using SYBR green in combination with gene-specific primers for human PLK1 (Forward: CTCAACACGCCTCATCCTC, reverse: GTGCTCGCTCATGTAATTGC), PLK2 (Forward: TGAGGAATAATTTTTGCGGC, reverse: AGATCTCGCGGATTATCGTC), PLK3 (Forward: TGTGCTCTTCAACGATGGC, reverse: GCTTTGTGCTGGTGGGATT) and PLK4 (Forward: CCTTCTGCAAATCTGGATGG, reverse: ACAGTGGTTTGGGAATCTGC). The samples were analyzed on a real time cycler (Applied Biosystems; 7500 fast).

Statistical analysis

For functional analyses, data was analyzed using Excel 2010 (Microsoft) and GraphPad Prism 6.0 (GraphPad Software, Inc.). p-values were determined using an unpaired t test, as indicated. p-values < 0.05 were generally considered statistically significant and are denoted by * throughout.

**Supplemental Figures and Tables**

Supplemental Figure S1

Mono-cytes

PBL

PMN

Fig. S1: Gating strategy for whole blood analysis. All cells were first gated (excluding debris, 1^st^ panel), then singlets (2^nd^ panel), then live cells (Aqua LiveDead stain negative, 3^rd^ panel), then individual populations as indicated (4^th^ panel). A representative donor is shown. PMN = polymorphonuclear neutrophils, PBL = peripheral blood lymphocytes.

**Supplemental Table 1: Properties of localized phospho-site S144:**

| Position | 144 |
| --- | --- |
| Localization probability | 0.91885 |
| PEP-score | 7.14 * 10^-8^ |
| Modified Sequence | _MATFPGPGSS(ph)PAR_ |
| M/Z | 678.2943 |
| MaxQuant Version | 1.2.2.9 |

**Literature cited in Supplemental Information**

1 Wang, H. *et al.* A frequent hypofunctional IRAK2 variant is associated with reduced spontaneous hepatitis C virus clearance. *Hepatology* **62**, 1375-1387, doi:10.1002/hep.28105 (2015).

2 Wang, H. *et al.* A coding IRAK2 protein variant compromises Toll-like receptor (TLR) signaling and is associated with colorectal cancer survival. *J Biol Chem* **289**, 23123-23131, doi:10.1074/jbc.M113.492934

M113.492934 [pii] (2014).

3 Motshwene, P. G. *et al.* An oligomeric signalling platform formed by the toll-like receptor signal transducers MyD88 and IRAK4. *J Biol Chem*, doi:M109.022392 [pii]

10.1074/jbc.M109.022392 (2009).

4 Dossang, A. C. *et al.* The N-terminal loop of IRAK-4 death domain regulates ordered assembly of the Myddosome signalling scaffold. *Sci Rep* **6**, 37267, doi:10.1038/srep37267

srep37267 [pii] (2016).

5 Boersema, P. J., Raijmakers, R., Lemeer, S., Mohammed, S. & Heck, A. J. Multiplex peptide stable isotope dimethyl labeling for quantitative proteomics. *Nat Protoc* **4**, 484-494, doi:10.1038/nprot.2009.21

nprot.2009.21 [pii] (2009).

6 Cox, J. & Mann, M. MaxQuant enables high peptide identification rates, individualized p.p.b.-range mass accuracies and proteome-wide protein quantification. *Nat Biotechnol* **26**, 1367-1372, doi:10.1038/nbt.1511

nbt.1511 [pii] (2008).

7 Cox, J. *et al.* Andromeda: a peptide search engine integrated into the MaxQuant environment. *J Proteome Res* **10**, 1794-1805, doi:10.1021/pr101065j (2011).

8 Klimosch, S. N. *et al.* Functional TLR5 genetic variants affect human colorectal cancer survival. *Cancer research* **73**, 7232-7242, doi:10.1158/0008-5472.CAN-13-1746 (2013).
